# Impact of early detection of acute invasive fungal rhinosinusitis in immunocompromised patients

**DOI:** 10.1186/s12879-019-3938-y

**Published:** 2019-04-05

**Authors:** Mariana L. C. Silveira, Wilma T. Anselmo-Lima, Francesca M. Faria, Danielle L. C. Queiroz, Rodrigo L. Nogueira, Marcelo G. J. Leite, Ricardo M. Lessa, Belinda P. Simões, Edwin Tamashiro, Fabiana C. P. Valera

**Affiliations:** 1 Department of Ophthalmology, Otorhinolaryngology, and Head and Neck Surgery, Ribeirão Preto Medical School, University of São Paulo, Av. Bandeirantes, 3900 – 12° andar., São Paulo, São Paulo CEP: 14049-900 Brazil; 2 Division of Pathology, Clinics Hospital of Ribeirão Preto Medical School, University of São Paulo, São Paulo, Brazil; 3 Department of Internal Medicine, Division of Hematology, Ribeirão Preto Medical School, University of São Paulo, São Paulo, Brazil

**Keywords:** Rhinosinusitis, Acute invasive fungal rhinosinusitis, Fungi, Frozen-section biopsy, Paraffin-embedded paraffin, Accuracy, Mortality rate

## Abstract

**Background:**

Early diagnosis of acute invasive fungal rhinosinusitis (AIFRS) is vital to improving outcomes in immunocompromised patients. This study evaluated the impact of a systematic protocol with nasal endoscopy and biopsies to early detect AIFRS in immunocompromised patients. Additionally, we compared the accuracy of frozen-section biopsy and culture with formalin-fixed paraffin-embedded (FFPE) biopsy.

**Methods:**

Retrospective cohort in a Tertiary Referral Hospital. Patients with the suspected diagnosis of AIFRS were evaluated following a standardized protocol, including serial nasal endoscopies and biopsies when necessary. The sensitivity and specificity of frozen-section biopsy and culture were also compared with FFPE.

**Results:**

The mortality rate related to AIFRS of this standardized cohort (13/43) was 30.2%. Better outcomes were observed in patients with disease limited to the turbinates and in those with higher peripheral neutrophils count. Frozen-section biopsy positivity correlated with FFPE findings for fungi detection (*p-value* < 0.0001), with a sensitivity of 90.6%, specificity of 72.7%, and accuracy of 86.0%.

**Conclusion:**

Implementation of this standardized protocol was related to a considerably low mortality rate among patients with suspected AIFRS at our Institution. Frozen-section biopsy revealed high accuracy to diagnose AIFRS. The current protocol including frozen-tissue biopsy improved the evaluation and survival rates of immunocompromised patients with presumed AIFRS.

## Background

Acute invasive fungal rhinosinusitis (AIFRS) is a life-threatening disease, affecting mostly immunocompromised patients with neutrophilic dysfunction [1–3]. In these patients, saprophytic fungi, particularly *Zygomycetes* and *Aspergillus*, can invade the nasal mucosa and blood vessels, leading to rapid dissemination into the orbits, palate and the brain [2–4]. For this reason, a systematic review states that the overall survival rate of AIFRS patients is as low as 50% [2]. Early diagnosis and immediate treatment, including antifungal therapy and surgical debridement, are considered vital for better survival rates [2].

Unfortunately, patients with severe neutropenia usually present with vague symptoms (such as fever lasting for more than 48 h) in early stages of AIFRS [5]. Other signs such as facial swelling or pain, proptosis, headache, seizures, or focal neurologic alterations are indicative of a later progression of this disease [2, 5], ultimately with poor prognosis [2, 6]. Nasal endoscopy can reveal crusting, pale mucosa, or necrosis in the affected areas [6, 7].

Imaging workup is essential to evaluate extra-sinonasal extensiveness of the disease and for surgical planning. CT scans and MRI are necessary to evaluate bone and soft tissue involvements (i.e., orbit/brain), respectively [2, 8], although their findings in early stages are usually non-specific [7]. Unilateral disease and bone thickening on CT scans are highly suggestive of AIFRS [6]. Notably, some imaging tests are completely normal despite the endoscopic findings suggestive of AIFRS [9].

Formalin-fixed paraffin-embedded (FFPE) histopathological exam, including hematoxylin-eosin (HE) and Gomori methenamine-silver (GMS) staining, is the most reliable test to confirm fungi invasion into the tissue [3, 8, 9]. However, processing for FFPE is time-consuming, which contrasts with the urgency to diagnose AIFRS. To obtain faster results, some groups have advocated the inclusion of frozen-section biopsy [5, 9–12] in the evaluation of patients at risk. In most of these studies, frozen-section biopsy during surgery has demonstrated moderate to high sensitivity and specificity when compared with FFPE histopathology.

In a previous case-series from our service [6], we observed a mortality rate of 50% in 32 AIFRS patients. To improve our outcomes, we have implemented a new protocol for evaluation of neutropenic patients, which included serial nasal endoscopy (every 48 h), associated with biopsy whenever appropriate.

The objective of this study was to investigate the impact of this protocol in the diagnosis and mortality rate of AIFRS, as well as to assess the sensitivity and specificity of frozen section biopsy in comparison with FFPE histopathology.

## Methods

This is a retrospective study performed in a tertiary referral hospital (Clinics Hospital – Ribeirão Preto Medical School, University of São Paulo, Brazil). All patients with the suspected diagnosis of AIFRS were evaluated by the ENT team between January 2010 and December 2015.

**Fig. 1:**
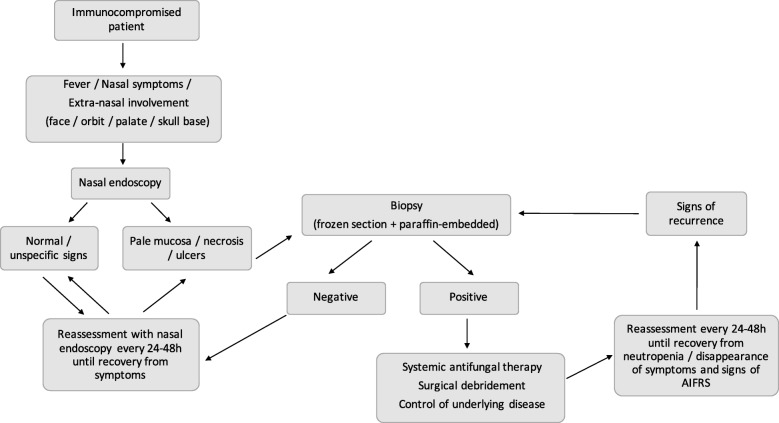
Algorithm of evaluation of an immunocompromised patient with suspected AIFRS

In 2010, after a multidisciplinary meeting, (composed predominantly by ENTs and hematologists) we have implemented a more comprehensive protocol to screen AIFRS in patients under risk. Every immunocompromised patient with persistent fever of unknown origin (48 h or more, not responding to antibiotic therapy); sinonasal symptom – rhinorrhea, nasal obstruction, epistaxis, crusting; and/ or facial edema - was immediately referred from hematologists to the ENT team. All these patients underwent thorough physical examination, nasal endoscopy, complete blood count (CBC) and sinonasal CT scan assessment (Fig. 1).

CT scans were scored according to a binary protocol (based on Lund-Mackay score), scoring “0” when the region was normal, and “1” if there was mucosal or bone thickening. The following sites were evaluated: maxillary sinus, anterior ethmoidal cells, posterior ethmoidal cells, frontal sinus, sphenoid sinus, and nasal cavity, with a possible maximum score of 12 points (6 points for each side).

Nasal endoscopy was performed in all patients, and we scored as “0” when normal or “1” when any suspicious mucosal alteration (pale area, darkened tissue, crust, ulcer or hyphae) was observed. Areas systematically evaluated during the nasal endoscopy were nasal septum, middle and inferior turbinates, lateral nasal wall, and nasopharynx. If any mucosa alteration was detected, three 5-mm specimens of the suspicious area were collected using nasal punch forceps: one for frozen-section biopsy, one for routine FFPE analysis (HE and GMS staining) and the third specimen for fungal culture. As routine in our service, complete blood count is performed daily in these patients. In severe thrombocytopenic patients (those with less than 20,000 platelets/ml), nasal endoscopy and biopsy were performed immediately after platelet transfusion. In all other patients, biopsies were obtained without major complications. Some patients received cotton embedded with vasoconstrictor to contain local bleeding.

**Fig. 2:**
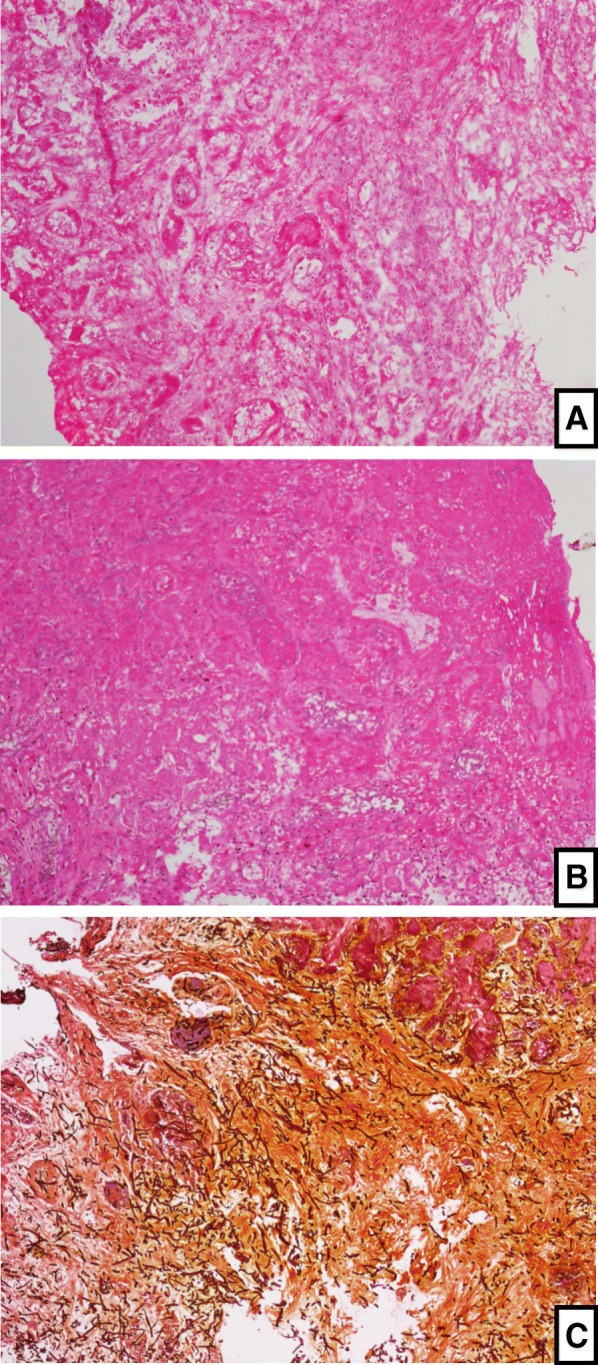
Histological sections of a patient with AIFRS, showing extensive necrosis, and abundance of hyphae invading mucosa with vascular embolization. Paucity septation with predominant acute angle division suggests *Aspergillus* species. 2**a**) Frozen-section section hematoxylin-eosin stain, 100x; 2**b**) Paraffin-embedded slide, hematoxylin-eosin stain, 100x; 2**c**) Paraffin-embedded slide, Gomori-methanamine-silver stain, 100x

Two samples were immediately sent to the Pathology Laboratory. The first was submitted to frozen-section biopsy, following a standardized protocol. Briefly, the specimen was immersed in Tissue-Tek medium at Freezing Microtome (Microm-HM-525), at -28 °C. Histological sections of 4–5 μm were obtained, colored with Hematoxylin-Eosin, and evaluated with an Olympus-BX51 microscope, to assess the presence of fungi. The mean time for this analysis was about 30 min (Fig. 2a).

The other sample was submitted to FFPE testing. The sample was fixed in 10% buffered-formalin and processed by an automatic tissue processor. The specimens embedded in paraffin were sectioned (4–5 μm), stained with HE and GMS, and analyzed for fungal presence and invasiveness of the mucosa. The time for this analysis in our service was about 5–7 days (Fig. 2b).

The third sample was sent to the mycology laboratory and cultured on *Sabouraud* medium. The mean time for a positive result was 7 days.

Patients with positive nasal biopsies (confirmed AIFRS) or those with highly suggestive symptoms/signs of AIFRS underwent surgical debridement and systemic antifungal therapy (Fig. 1). Surgical debridement was mainly performed by endoscopic sinus surgery (ESS), associated with open surgeries whenever orbital, intracranial, or palatal involvement was observed. During this procedure, new samples were obtained for frozen-section, FFPE biopsies, and micology analysis. Immediately after diagnosis, systemic liposomal amphotericin-B or voriconazole was started. Patients were then periodically evaluated at the ENT clinic with nasal endoscopy (every 24–48 h) until neutropenia was recovered and no sinonasal symptoms and signs were present (Fig. 1).

Data regarding nasal endoscopic findings, associated comorbidities, CBC, CT scan evaluation, biopsy, and culture were obtained from medical records. Patient outcomes were assessed to calculate mortality rate related to the fungal disease. Patients with incomplete clinical data were excluded from the study.

This study was approved by the local IRB (Clinics Hospital – Ribeirão Preto Medical School – University of São Paulo), under the number of CAAE 62294116.6.0000.5440. As this was a retrospective cohort, and data was collected from medical records, exemption to obtain informed consent was demanded to local IRB, and it was considered approved.

### Statistical analysis

The mortality rate from the cohort was described, considering the disease-related mortality, i.e. the unfavorable outcome when patients died explicitly from invasive fungus infection either sinonasal or pulmonary.

The clinical factors of both groups (surviving patients vs. dead ones) were compared using the unpaired Student t-test or with Fisher’s exact test. Fisher’s exact test was also used to evaluate the association between FFPE biopsy with frozen section biopsy and culture. Sensitivity, specificity, positive predictive value (PPV), and negative predictive values (NPV) were assessed, considering both frozen-section biopsy and culture as index tests, and paraffin-embedded biopsy as the reference standard test [13]. For all the statistical analyses, difference was considered significant when *p-value* < 0.05.

## Results

Forty-six patients with the suspected diagnosis of AIFRS were evaluated during the period. Three patients were excluded because they did not have complete data in their files.

Among the remaining 43 patients, 23 were male; and age ranged from 1 to 70 years (mean: 40.1 ± 20.4). The following associated diseases were present: hematological malignancies in 25 patients (58.2%); aplastic anemia in 11 (25.6%), autoimmune disorders in 3 (7%), diabetes mellitus in 2 (4.6%), HIV infection in 1 (2.3%) and chronic renal failure in 1 (2.3%).

Thirteen patients (30.2%) died of invasive fungal infection: in 11 cases, AIFRS was the cause of death; another two died due to pulmonary Aspergillosis. The associated diseases among the patients that died were hematological malignancies (*n* = 8), aplastic anemia (*n* = 3), diabetes (*n* = 1), and autoimmune disorder (*n* = 1). The distribution of mortality regarding the primary disease was not significant (*p-value* = 1.00).

Among the patients with hematological diseases (aplastic anemia or hematological malignancies, *n* = 36), the average time of neutropenia when AIFRS was diagnosed was 35 days. These patients exhibited white blood cell count of 850 ± 800cells/μL and absolute neutrophil count of 330 ± 200cells/μL. The remaining seven patients had a different primary disease, without history of neutropenia or decreased white blood cell count at the evaluation.

Neither the length of neutropenia (45.7 ± 18 vs. 33.8 ± 8 days, *p-value* = 0.55) nor the absolute white blood cell count (1.1 ± 0.2 vs. 0.7 ± 0.3cells/μL, *p-value* = 0.48) was different between the groups who lived and the one that died, respectively. Nonetheless, the level of neutropenia was significantly lower in patients who died (0.2 ± 0.1 cells/μL) when compared with the patients alive (1.8 ± 0.7 cells/μL, *p-value* = 0.04).

During the first nasal endoscopy, 31 patients presented pale or darkened mucosa only in the middle or inferior turbinates. Among them, eight patients died (mortality rate = 25%). In contrast, 7 of 12 patients who presented with fungal disease in the lateral wall or nasal septum died (mortality rate = 58%; *p* = 0.07).

On CT scan, the most affected areas were the middle meatus, ethmoid, and the maxillary sinus (respectively in 31, 25 and 27 patients). Frontal and sphenoid sinuses were affected in 14 patients. Five patients presented extra-nasal extension on CT scans, four in orbit and one intracranially. Disease extension on CT scan was not related to the mortality rate (*p* = 0.47). Notably, five patients (11.6%) presented a total CT scan score of “0”, meaning that they did not have any sinonasal change on CT scans. Additionally, other 12 patients (27.9%) presented a total score of “1” or “2”, indicating minimal changes on CT scans.

Thirty-five patients presented positive biopsies at ENT evaluation, undergoing surgery as soon as medical conditions were allowed. In 8 patients, both frozen and paraffin-embedded biopsies were negative, and they were followed-up with nasal endoscopy every 24–48 h. In 6 of them, symptoms and signs were controlled clinically, with no confirmation of AIFRS; other two patients persisted with fever and changes in nasal endoscopy, and surgery was indicated regardless the initial biopsies. Surgery performed was the endoscopic debridement of all involved areas in sinonasal areas (37 patients), associated with orbital exenteration in 4 patients and intracranial debridement in 1 patient.

After surgery, all patients were submitted to scheduled evaluation with nasal endoscopy. As soon as a pale/necrotic area were observed, they were reassessed with a new ESS debridement. Fourteen patients (32.5%) underwent multiple procedures. The number of surgeries for those patients who died was similar with those who were alive (respectively 1.7 ± 0.3 vs. 1.3 ± 0.2, *p-value* = 0.20).

The positivity-rate in both frozen-section and FFPE biopsies are shown in Table 1. The sensitivity of frozen-section biopsy was 90.6%; specificity was 72.7%; PPV was 90.6%; NPV was 72.7%; and accuracy was 86.0% (*p-value* < 0.0001).

**Table 1:** Distribution of patients (*n* = 43) regarding the positivity rate in frozen-section and formalin-fixed paraffin-embedded (FFPE) biopsies

	Positive FFPE biopsy	Negative FFPE biopsy
Positive Frozen-section biopsy	29	3
Negative Frozen-section biopsy	3	8

Data comparing culture with FFPE biopsy were available in 29 patients. Fungal culture was positive in 6 cases for *Aspergillus ssp.*, 3 for *Mucor*, and 1 for *Histoplasma sp*. The agreement between culture and FFPE biopsy was not significant (*p* = 0.62). Sensitivity of culture was 80.0%; specificity was 40.0%; PPV was 21.0%; NPV was 90.9%; and accuracy was 55.2% (Table 2).

**Table 2:** Distribution of patients (*n* = 29) regarding the positivity rate in culture and formalin-fixed paraffin-embedded (FFPE) biopsies

	Positive FFPE biopsy	Negative FFPE biopsy
Positive Culture	10	1
Negative Culture	12	6

The mortality rate of this current standardized cohort was 30.2% (*n* = 13/43).

## Discussion

The early diagnosis of AIFRS is challenging and demands accurate and fast tests [14]. With this aim, we have established a protocol to maximize the sensitivity to diagnose AIFRS, in such a way that patients under risk, with subtle or even with non-specific symptoms, are immediately referred by hematologists to ENT evaluation. During this exam, to increase sensitivity in the diagnosis, we have elected nasal endoscopy as the essential exam, followed by biopsy, instead of CT findings.

Actually, as we observed in our current cohort, a significant percentage of patients with confirmed AIFRS (39.5%) had CT scans with few or even normal findings. Our data reinforce that nasal endoscopy must be elected as the preferred choice to evaluate patients at risk because it can reveal subtle changes in the nasal mucosa [6, 14, 15] that are not detected by imaging exams [6, 15]. Taking into consideration only CT scans findings, these patients would have experienced a delayed treatment, and this could negatively impact on their outcome.

After the implementation of this protocol, we have been able to diagnose AIFRS in a faster and more accurate way. As a consequence, patients are undergoing surgery in early stages of the disease, with restricted sinonasal involvement. For instance, the majority of cases (72.1%) had invasiveness restricted to the turbinates, mainly the middle turbinate. The combination of limited invasiveness of the disease at the diagnosis with a careful post-operative follow-up was related to a good outcome (30.2% of disease-related deaths) when compared to the literature [2, 6, 12, 14, 15, 18].

Another contribution to detect AIFRS in early stages was the performance of frozen-section biopsies [3, 4, 10, 15, 16], the presence of artifacts during the freezing processing is still concerning. In our cohort, we observed that frozen-section and paraffin-embedded biopsies were well correlated (accuracy of 86%). Our accuracy is quite similar to previous reports in literature [5, 9–11], showing high sensitivity and moderate specificity. However, three patients in our cohort presented positive frozen-section but negative paraffin-embedded biopsy, and this might have occurred because in our study small samples were taken under local anesthesia in awake patients for the frozen-section and paraffin-embedded biopsies, while in other studies they were obtained intra-operatively [5, 9, 10]. To the best of our knowledge, the present study is the largest series of patients that specifically used frozen-section biopsy for the diagnosis of AIFRS.

Mycological tissue culture has also been considered helpful in evaluating AIFRS because it can determine the fungi species and direct the antifungal therapy. However, it does not show invasiveness of mucosa and, therefore, cannot establish the definitive diagnosis of AIFRS. Moreover, it usually takes a minimum of 7 days to obtain the results, and a significant number of negative results is expected [10, 17]. For instance, only 11 patients (36%) in our recent cohort had positive culture results. The association between culture results and paraffin-embedded biopsy was not significant, with low accuracy of 55.2%.

The cause of immunodeficiency was not related to prognosis in our cohort and this may be due to the limited number of patients without hematological diseases. In this specific population, the low number of neutrophils was negatively related to the survival rate. This agrees with several other studies [2, 14, 18], pointing out that neutropenia might be one of the most critical risk factors for the development and prognosis of AIFRS.

The surgical procedure associated with prompt initiation of antifungal therapy is critical for the outcome of AIFRS. In our protocol, patients were carefully followed-up with periodic nasal endoscopy after surgery, and every new alteration was determinant to indicate a further surgical debridement. In our series, we observed that 32.5% of patients needed at least one revisional surgery, similarly as reported by Payne et al. [14]. As important as the surgery itself, we believe that the continuous follow-up might have contributed to the better outcomes in these patients [2].

In summary, we observed that frozen-section biopsy is a fast and reliable exam to confirm the diagnosis of fungal invasion, with good accuracy when compared to the gold-standard FFPE biopsy.

Most importantly, the present protocol, based on direct suspicion by hematologists, comprehensive screening by ENTs and follow-up with nasal endoscopy in patients under risk, was associated with a considerable decrease in our Institutional mortality rate related to AIFRS, from 50 to 30.2%. This algorithm reinforces that nasal endoscopy should be preferred to CT scans to identify AIFRS since almost 40% of the patients in this cohort presented normal CT images. Finally, the constant pursuit of a precise AIFRS identification, either at primary diagnosis or at recurrence, is essential for improving outcome in immunocompromised patients.

## Conclusions

The present protocol, based on an immediate evaluation by ENTs in patients under risk of AIFRS, was associated with a considerable decrease in mortality rate related to AIFRS in our hospital. Also, we observed that frozen-section biopsy is a fast and reliable exam to confirm the diagnosis of fungal invasion, with good accuracy when compared to the gold-standard FFPE biopsy.

## Abbreviations

AIFRS: Acute invasive fungal rhinosinusitis
ENT: Ear nose and throat specialist
FFPE: Formalin-fixed paraffin-embedded
